# Modulation of Mouse Coagulation Gene Transcription following Acute *In Vivo* Delivery of Synthetic Small Interfering RNAs Targeting HNF4α and C/EBPα

**DOI:** 10.1371/journal.pone.0038104

**Published:** 2012-06-01

**Authors:** Huma Safdar, Ka Lei Cheung, Hans L. Vos, Frank J. Gonzalez, Pieter H. Reitsma, Yusuke Inoue, Bart J. M. van Vlijmen

**Affiliations:** 1 Einthoven Laboratory for Experimental Vascular Medicine, Department of Thrombosis and Hemostasis, Leiden University Medical Center, Leiden, The Netherlands; 2 Laboratory of Metabolism, National Cancer Institute, Bethesda, Maryland, United States of America; 3 Department of Chemistry and Chemical Biology, Graduate School of Engineering, Gunma University Kiryu, Gunma, Japan; Center of Ophtalmology, Germany

## Abstract

Hepatocyte nuclear factor 4α (HNF4α) and CCAAT/enhancer-binding protein α (C/EBPα) are important for the transcriptional control of coagulation factors. To determine *in vivo* the direct role of HNF4α and C/EBPα in control of genes encoding coagulation factors, a synthetic small interfering (si)RNA approach was used that enabled strong reduction of mouse hepatic HNF4α and C/EBPα under conditions that minimized target-related secondary effects. For both HNF4α and C/EBPα, intravenous injection of specific synthetic siRNAs (siHNF4α and siC/EBPα) resulted in more than 75% reduction in their liver transcript and protein levels 2 days post-injection. For siHNF4α, this coincided with marked and significantly reduced transcript levels of the coagulation genes *Hrg*, *Proz*, *Serpina5*, *F11*, *F12*, *F13b*, *Serpinf2*, *F5*, and *F9* (in order of magnitude of effect) as compared to levels in control siRNA injected animals. Significant decreases in HNF4α target gene mRNA levels were also observed at 5 days post-siRNA injection, despite a limited level of HNF4α knockdown at this time point. Compared to HNF4α, C/EBPα knockdown had a modest impact on genes encoding coagulation factors. A strong reduction in C/EBPα transcript and protein levels resulted in significantly affected transcript levels of the control genes *Pck1* and *Fasn* and a modest downregulation for coagulation genes *Fba*, *Fbg* and *F5*. *F5* and *F11* were the sole coagulation genes that were significantly affected upon prolonged (5 day) C/EBPα knockdown. We conclude that in the mouse, HNF4α has a direct and essential regulatory role for multiple hepatic coagulation genes, while a role for C/EBPα is more restricted. In addition, this study demonstrates that synthetic siRNA provides a simple and fast means for determining liver transcription factor involvement *in vivo*.

## Introduction

Hepatocyte nuclear factor 4α (HNF4α) and CCAAT/enhancer-binding protein α (C/EBPα) are two distinct transcription factors that are of key importance in controlling many genes specifically expressed in the liver and associated with a number of critical metabolic pathways [1]–[3]. In addition, both transcription factors are claimed to be critical for control of the hepatic genes encoding proteins in the coagulation pathway. For HNF4α, first evidence came from *in vitro* gene promoter studies for human coagulation genes. Functional HNF4α binding sites were identified near the genes encoding factor (F) II (F2), VII (F7), VIII (F8), IX (F9), X (F10), XI (F11), XII (F12), protein S (PROS1), protein Z (PROZ) and antithrombin III (SERPINC1) [4]–[13].

The *in vivo* importance of HNF4α in regulating hepatic transcription of coagulation genes described in studies using hepatocyte-specific HNF4α knockout mice [14], [15]. *Hnf4a* disruption affected expression of factor (F) *F5*, *F9*, *F11*, *F12*, *F13b*, protein C inhibitor (*Serpina5*), protein Z (*Proz*), α2-antiplasmin (*Serpinf2*), protein Z inhibitor (*Serpina10*), and heparin Cofactor II (*Serpind1*), whereas no effects were observed for *F2*, *F7*, *F8*, *F10*, protein S (*Pros1*) and antithrombin (*Serpinc1*) [14], [15]. For C/EBPα, studies on the relevance to coagulation are limited. A recent CHIPseq study determined the genome-wide occupancy of C/EBPα in the livers of human, mouse, dog, opossum, and chicken. Fibrinogen A (FGA) and F2 were identified among 32 genes located near 35 C/EBPα binding events that were conserved among these five vertebrates [16]. In the mouse genome, *Fga* and *F2*, C/EBPα binding sites were 45 and 64 bp from the transcription start site, respectively. However, it should be emphasized that binding does not necessarily correlate with functional activity in controlling gene transcription. Furthermore, carriers of hemophilia B Leiden have a causal mutation in a C/EBPα binding site in F9 promoter [17], and in line, liver-specific C/EBPα-null mice display reduced hepatic expression of *F9* [17], [18]. Finally, *in vitro* studies demonstrated requirement of C/EBPα for F8 expression, but this involved nonhepatic inflamed cells [19]. Whether other coagulation genes other than FGA, F2, F9 and F8 are regulated by C/EBPα is unknown.

The study of the *in vivo* roles of HNF4α and C/EBPα in control of gene transcription in liver employed a conditional gene knockout approach because conventional gene knockouts for HNF4α and C/EBPα were embryonic lethal [20], [21]. In general, a conditional gene knockout approach does not allow a rapid significant deletion of the gene of interest *in vivo* without challenging liver physiology; fast adenovirus-mediated hepatic delivery of the required Cre- recombinase does allow rapid hepatic disruption of ‘floxed’ alleles but is concomitant with adenovirus-related acute hepatic inflammation [22], [23]. Similarly, inducible liver-specific gene disruption based on the MX1-Cre transgene requires a burst of circulating interferon to evoke the necessary activation of MX1-Cre [24]. Meaningful studies of the role of hepatic transcription factors can therefore only start after weaning of the adenovirus or interferon effects. At that time, transcription factor deletion may already have induced (secondary) changes in liver physiology and compensatory changes in expression of other hepatic transcription factors. It may lead to a possible misinterpretation of the direct role of a given transcription factor in gene regulation. Hence, the current observations regarding the role of HNF4α and C/EBPα in regulating coagulation gene transcription obtained in adult mice lacking HNF4α and C/EBPα from birth on (Cre recombinase under control of the albumin promoter) [25], following Cre supplied by means of adenovirus [26] or the MX1-Cre transgene [27] may be in part secondary to changes in liver physiology and changes in expression of other hepatic transcription factors. This may, explain the unexpected transcriptional increase of numerous hepatically expressed genes including the coagulation gene *Serpind1* and absence of effects for the *in vitro* identified targets *F2*, *F7*, *F8*, *F10*, *Pros1* and *Serpinc1* in livers from mice lacking HNF4α from birth [14], [15].

Recently, lipid-based reagents became available that allow efficient delivery of synthetic small interfering (si) RNAs to livers of adult mice following systemic injection [28]. Thus, transient knockdown of target gene expression can be achieved rapidly (within two days post siRNA delivery) and does not involve changes in liver physiology as a result of harsh methodology. In the present study, we used this *in vivo* siRNA approach to rapidly reduce HNF4α and C/EBPα expression in mouse livers and to determine the impact of these two distinct transcription factors on hepatic coagulation gene transcription.

## Materials and Methods

### siRNA screening and validation

Pre-designed siRNAs for mouse HNF4α and C/EBPα mRNAs were purchased from Ambion Applied Biosystems, Carlsbad, California, USA (Ambion Silencer® Pre-designed for HNF4α; catalogue numbers 67633 (#1), 67634 (#2) and 67635 (#3) with sense sequences GGC AGA UGA UCG AAC AGA UUU, CCA AUG UCA UUG UUG CUA AUU, and AGA GGU CCA UGG UGU UUA AUU, respectively and for mouse C/EBPα; catalogue numbers 63853 (#1), 63854 (#2), 63855 (#3) with sense sequences GCA AAA AUG UGC CUU GAU AUU, AAA GCU GAG UUG UGA GUU AUU, and ACU CAA AAC UCG CUC CUU UUU, respectively). Ambion's siNEG (catalogue number 4404020) was used as control siRNA. This negative control siRNA was selected using a modified blast to account for short sequence length and demonstrated to exclude significant homology to any known gene targets in RefSeq and MirBase (more detailed documentation on this negative control siRNA is available on the manufacturer's website [29]). Hepatocytes from female C57Black6/J mice (Charles River, Maastricht, The Netherlands) were isolated through retrograde collagenase perfusion and cultured in collagen-coated dishes exactly as previously described [30]. Twenty four hours after isolation 10^6^ cells were transfected with the siRNA (final concentration 0.3, 3 or 30 nM) using the Dharmafect Duo transfection reagent® (Dharmacon, T-2010-03) according to the manufacturer's protocol. Twenty-four hours after transfection, levels of HNF4α and C/EBPα mRNAs were determined by quantitative real-time PCR (see below). Sequences from siRNAs yielding maximal reduction of transcript levels at a siRNA concentration of 3 nM were considered for use in *in vivo* studies.

### Gene knockdown in mouse liver

Control siNEG, siHNF4α and siC/EBPα (Ambion In-Vivo-Ready for catalogue numbers 4404020, 67633 and 63855, respectively) were complexed with Invivofectamine® 2.0 Reagent (Invitrogen, Life technologies Corporation, USA) exactly according to the manufacturer's protocol. Subsequently, female C57Black/6J mice (weighing 17–19 gram) were intravenously injected via the tail vein with 200 µl complexed siRNA at a dose of approximately 7 mg of siRNA per kg body weight (in total 54 animals, 18 animals per siRNA). At two and five days post siRNA injection, animals (9 mice per siRNA for each time point) were anesthetized by a subcutaneous injection with a mixture of ketamine (100 mg/kg), xylazine (12.5 mg/kg) and atropine (125 µg/kg) after which the abdomen was opened by a midline incision and a blood sample on sodium citrate (final concentration 0.32%) was drawn from the inferior caval vein. Plasma was obtained by centrifugation and stored at −80°C until use. Liver was isolated and weighed, and liver left lobule was snap-frozen for mRNA and protein analyses and stored at −80°C until use. All mice were housed under a 12-h light/dark cycle, with standard chow diet and drinking water provided ad libitum. All experimental procedures were approved by the animal welfare committee of the Leiden University (under registration # 11005).

For HNF4α, as a reference, liver materials from 45-day old female HNF4α-null mice with a liver-specific deletion of exons 4 and 5 of the *Hnf4a* gene (HNF4α-floxed/floxed with albumin-Cre; KO) or control mice (HNF4α-floxed/floxed without albumin-Cre; FLOX) [25] were used.

### RNA isolation and real-time RT-PCR

Liver samples (20–30 mg) were homogenized in RNAzol (Tel-Test) and RNA isolation and cDNA synthesis was performed as previously described [31]. Gene-specific quantitative real-time PCR (QPCR) primers for *Pck1*, *Fasn*, *Scd1*, *Lgp* and *Gys2* have been described previously [32]; all other gene specific QPCR primers were designed with the Primer Express software (Applied Biosystems). QPCR primer sequences are presented in Table 1. QPCR was performed on the ABI Prism 7900 HT Fast Real-Time PCR System from Applied Biosystems and data were analysed using the accompanying Sequence Detection System software. The comparative threshold cycle method with β-actin as internal control was used for quantification and normalization. siNEG-injected animals were set as a reference and the ΔCt values of the individual samples were related to the mean ΔCt of the reference group.

**Table 1:** QPCR primer sequences.

Gene	Forward primer (5′- 3′)	Reverse primer (5′- 3′)
*Actb*	AGGTCATCACTATTGGCAACGA	CCAAGAAGGAAGGCTGGAAAA
*Apoc2*	AAGATGACTCGGGCAGCCT	CAGAGGTCCAGTAACTTAAGAGGGA
*Apoa4*	CAGCTGACCCCATACATCCAG	TCATCGAGGTGTGCAGGTTG
*Cd36*	GTTCTTCCAGCCAATGCCTTT	ATGTCTAGCACACCATAAGATGTACAGTT
*Cebpa*	ATAGACATCAGCGCCTACATCGA	GTCGGCTGTGCTGGAAGAG
*Cebpb*	CGGGACTGACGCAACACA	CCGCAGGAACATCTTTAAGTGATTA
*Cyp4v3*	CTCTCCGAGTTTTCCCATCTGT	TTGTAACCGCCCACTTCACA
*F2*	GGACGCTGAGAAGGGTATCG	CCCCACACAGCAGCTCTTG
*F5*	CATGGAAACCTTACCGACAGAAA	CATGTGCCCCTTGGTATTGC
*F7*	CGTCTGCTTCTGCCTCTTAGA	ATTTGCACAGATCAGCTGCTCAT
*F9*	GCAAAACCGGGTCAAATCC	ACCTCCACAGAATGCCTCAATT
*F10*	GTGGCCGGGAATGCAA	AACCCTTCATTGTCTTCGTTAATGA
*F11*	GAAGGATACGTGCAAGGGAGATT	CAAGTGCCAGACCCCATTGT
*F12*	GGGCTTCTCCTCCATCACCTA	GCAACTGTTGGTTTTGCTTTCC
*F13a1*	GATGTCCTGGCCAAACAAAG	GGCAGCACCTCGGACCTT
*F13b*	GACACTGCCCCCTGAGTGTGTTGAAA	AACAACCACACCGTTTGCTATG
*Fasn*	CCCTTGATGAAGAGGGATCA	ACTCCACAGGTGGGAACAAG
*Fga*	TTCTGCTCTGATGATGACTGGAA	GGCTTCGTCAATCAACCCTTT
*Fgg*	TGCTGCCTGCTTTTACTGTTCTC	TCTAGGATGCAACAGTTATCTCTGGTA
*Gys2*	GACACTGAGCAGGGCTTTTC	GGGCCTGGGATACTTAAAGC
*Hnf4a*	AGAGGTTCTGTCCCAGCAGATC	CGTCTGTGATGTTGGCAATC
*Hrg*	AAAACGGATAATGGTGACTTTGC	TCCCCTCCTCTCGCTCTTATAA
*Lgp*	CCAGAGTGCTCTACCCCAAT	CCACAAAGTACTCCTGTTTCAGC
*Pck1*	CTGGCACCTCAGTGAAGACA	TCGATGCCTTCCCAGTAAAC
*Plg*	TGACATTGCCCTGCTGGAAAC	CAGACAAGCTGGAATGACTTTATCC
*Proc*	GCGTGGAGGGCACCAA	CCCTGCGTCGCAGATCAT
*Pros1*	GGTGGCATCCCAGATATTTCC	CACTTCCATGCAGCCACTGT
*Proz*	GCAGCCAGAGTCAGCCTAGCT	CACGCCGGCACAGAAGTC
*Scarb1*	GCCAGGAGAAATGCTTTTTGTT	GGCCTGAATGGCCTCCTTA
*Scd1*	AGCTGGTGATGTTCCAGAGG	GTGGGCAGGATGAAGCAC
*Serpina5*	TCTGGCATTACTGACCATACCAA	GACTCTTCAACCTCCATCATGGA
*Serpina10*	TGGCCCTGGAGGACTACTTG	CCATTTTCCTGGTTTTCATATTCTG
*Serpinc1*	TGGGCCTCATTGATCTCTTCA	CCTGCCTCCAGCAACGAT
*Serpind1*	GAATGGCAATATGTCAGGCATCT	CACTGTGATGGTACTTTGGTGCTT
*Serpinf2*	TTCTCCTCAACGCCATCCA	GGTGAGGCTCGGGTCAAAC

### Immunoblotting

Frozen liver material (10–20 mg) was grounded, liver protein (15 µg) was denatured, separated on 8–10% Novex® Tri-Glycine gels, and immunoblotted using a goat polyclonal IgG against human HNF4α (sc-6556, Santa Cruz Biotechnology) or rabbit polyclonal against rat C/EBPα (sc61, Santa Cruz Biotechnology). ß-actin was detected using rabbit polyclonal against human ß-actin (Ab8227, Abcam) and served as protein loading control. The antibodies are reactive to mouse HNF4α, C/EBPα (both p42 and p30 unit) and ß-actin, respectively. Bound IgG was detected using horseradish peroxidase-labeled anti-goat (sc-2020, Santa Cruz) or anti-rabbit (172-1019, Bio Rad) IgG followed by enhanced chemiluminescence system (Amersham Pharmacia Biotech) to detect peroxidase activity.

### Plasma analyses

Plasma alanine aminotransferase (ALT), aspartate aminotransferase (AST), alkaline phosphatase (ALP) and bilirubin (total and conjugated) levels were determined using routine clinical chemistry assays. Global coagulability of the plasma was determined by measuring the prothrombin time (PT, Simple Simon PT system, Zafena), and the activated partial thromboplastin time (APTT) by using the STA Neoplastin Plus reagent (Roche) on the STart 4 analyzer (Diagnostica Stago). Plasma F5 activity was analyzed by using chromogenic substrate conversion [33] and activity levels of factor (F) F11 and F12 were measured with APTT-based assays [31]. Plasma fibrinogen antigen levels were assessed with a commercial murine ELISA kit from Affinity Biologicals. In plasma assays of individual coagulation factors, pooled normal mouse plasma was used to generate standard curves and the control siNEG-injected group was set as a reference (100%).

### Statistical analyses

Data were analysed with the GraphPad Instat software. Statistical differences between control siNEG and siHNF4α or siC/EBPα groups were evaluated using a Mann-Whitney Rank sum test (animal studies) or Student's t-test (hepatocyte studies). A *P*-value of <0.05 was considered to be statistically significant.

## Results

### siRNA screening and validation

**Figure 1:**
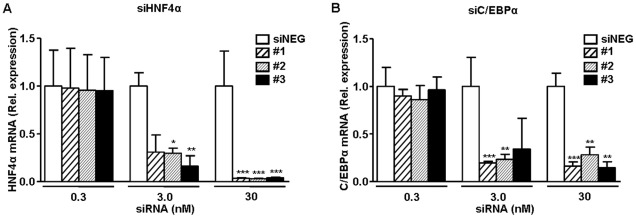
Screening and validation of siRNAs in mouse primary hepatocytes. Screening of siRNA in mouse primary hepatocytes 24 hours after transfection with 0.3, 3 and 30nM of three *Hnf4a*-specific (#1–3, panel A), three *Cebpa* -specific siRNAs (#1–3, panel B) or a control siRNA (siNEG, panel A and B, open bars). *Hnf4a* and *Cebpa* transcript levels were determined by quantitative real-time PCR. β-actin was used as internal control for quantification and normalization. The ΔCt values of the individual samples were related to the mean ΔCt of the reference group (siNEG). On the x-axis siRNA concentrations are indicated. Data are expressed as mean with error bars representing the difference between 2 POWER of upper and lower range of the mean ΔΔCt (difference 2∧ΔΔCt+SEM and 2∧ΔΔCt−SEM). Individual experiments were performed in triplicate. Data were statistically analyzed using the Student's t-test. *P*-values <0.05 were regarded as statistically significant. *P<0.05, **P<0.01, ***P<0.001.

**Figure 2:**
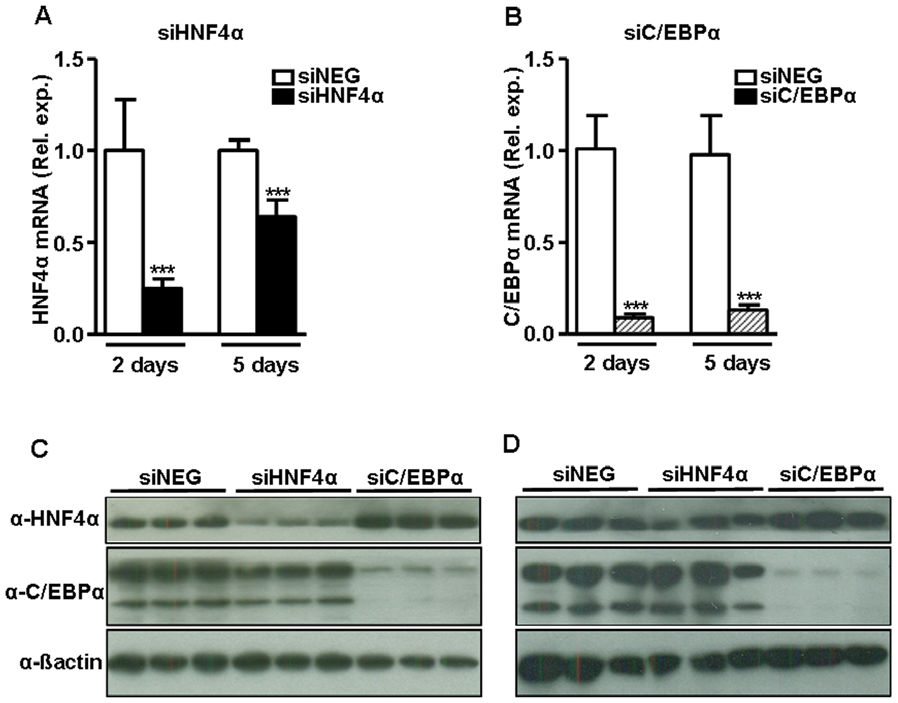
Knockdown of HNF4α and C/EBPα in mouse liver. The selected siHNF4α-specific siRNA #3 (67635), siC/EBPα-specific siRNA #1 (63853) and a control siNEG were subjected to large-scale preparation in the lipid-based in vivo transfection reagent optimized for hepatic delivery and intravenously injected in C57Black/6J mice (7 mg siRNA per kg mouse, injection volume 200 µl, 18 animals per siRNA). At two and five days post siRNA injection, mice (n = 9 per siRNA for both time points) were sacrificed and livers were subjected to HNF4α (panel A) or C/EBPα (panel B) transcript analysis by QPCR. β-actin was used as internal control for quantification and normalization. The ΔCt values of the individual samples were related to the mean ΔCt of the reference group (siNEG). Data are expressed as mean with error bars representing the difference between 2 POWER of upper and lower range of the mean ΔΔCt (see figure 1 legends). Data were statistically analysed using Mann Whitney Rank Sum test. *P*-values<0.05 were regarded as statistically significant. ***P<0.001. In addition, immunoblotting for HNF4α and C/EBPα was performed for liver homogenates that were prepared for three randomly selected mice per siRNA for both two days (panel C) and five days post injection (panel D). The C/EBPα antibody used, reacts with both C/EBPα p42 and p30.

To select an effective siRNA, mouse primary hepatocytes were transfected with three different predesigned synthetic siRNAs for HNF4α, and C/EBPα mRNAs. For HNF4α, all three siRNAs were highly and equally effective with over 95% reduction of *Hnf4a* transcript levels at a final concentration of 30 nM (Figure 1A). siRNA duplex #3 had the highest level of knockdown of *Hnf4a* transcript levels at the 3 nM concentration and was selected for use in *in vivo* experiments. For C/EBPα, all three siRNAs tested were highly effective, albeit that the levels of reduction (∼80%, Figure 1B) were somewhat lower as compared to the siRNAs for HNF4α. For C/EBPα siRNA duplex #1 was selected, being the most effective at 3 nM. The siHNF4α #3, siC/EBPα #1 and the siNEG were subjected to large-scale preparation in the lipid-based *in vivo* transfection reagent optimized for hepatic delivery, and injected intravenously into C57Black/6J female mice. Two days after injection, siHNF4α and siC/EBPα produced a more than 75% reduction in liver *Hnf4a* and *Cebpa* transcript levels (Figure 2A and 2B, respectively), as well as strongly reduced liver HNF4α and C/EBPα protein levels (Figure 2C). For siC/EBPα this strong level of knockdown of liver C/EBPα mRNA and protein persisted for at least five days (Figure 2B and 2D). For siHNF4α, HNF4α mRNA and protein levels remained reduced at five days, but, as quantified for the transcript, only at a mean level of 36% (Figure 2A and 2D). This relatively quick return to normal HNF4α levels for siHNF4α was also observed in a second independent experiment and could not be overcome by a repeat intravenous siHNF4α injection (7 mg/kg) at day 2 after the first injection (data not shown).

During the 5-day observation period siHNF4α and siC/EBPα did not affect mouse body weight and liver weight as compared to siNEG injected animals or uninjected controls. Gross pathological analysis revealed no abnormalities. Plasma bilirubin, ALT, AST and ALP in siNEG injected animals were comparable to those observed for uninjected controls (Table 2) as expected [28]. As compared to the siNEG injected controls, at two days post siRNA injection, ALP levels were significantly increased by siHNF4α, and at 2 days significantly reduced by siC/EBPα. At 2 and 5 days, plasma bilirubin, AST, ALT levels in siHNF4α and siC/EBPα were below the limits of detection and comparable to siNEG injected animals.

**Table 2:** Plasma analysis of siHNF4α and siC/EPBα injected mice.

	2 days post siRNA injection			5 days post siRNA injection		
	siNEG	siHNF4α	siC/EPBα	siNEG	siHNF4α	siC/EPBα
PT (sec)	11.4±0.1	11.4±0.2	11.6±0.1	12.2±0.1	11.8±0.1*	12.5±0.1*
aPTT (sec)	28.2±0.6	28.6±0.8	27.8±0.4	31.0±0.4	31.9±0.7	30.6±0.6
F5 (%)	100±7	80±11	90±5	100±7	126±6	83±5
F11 (%)	100±6	80±4*	N.D.	100±5	90±8	N.D.
F12 (%)	100±2	100±2	N.D.	100±4	85±8	N.D.
Fbg (%)	100±5	98±3	72±2**	100±10	121±8	70±3**
Tbil (µmol/L)	8.7±0.7	8.5±0.2	8.4±0.4	8.6±0.3	9.0±0.2	8.6±0.4
ALT (U/L)	<20	<20	<20	<20	<20	<20
AST (U/L)	43±8	31±4	32±2	34±3	40±4	39±5
ALP (U/L)	120±8	181±5**	98±4*	90±11	107±7	69±4

Data are represented as mean ± SEM with the group injected with negative siRNA set as a reference.

* *P*<0.05 and

** *P*<0.01 versus mice injected with control siNEG. Prothrombine time (PT), activated partial thromboplastine time (aPTT), Factor activity (F11), Factor 12 activity (F12), Fibrinogen antigen (Fbg) and circulating liver enzymes alanine transaminase (ALT), aspartate aminotransferase (AST), alkaline phosfatase (ALP) and plasma total billirubine (Tbil). N.D. = not determined. Values for uninjected control mice: billirubin 8.3±0.2 µmol/L, ALT<20 U/L, AST 28±3 U/L, ALP 96±6 U/L.

### Changes in transcription of control genes following liver HNF4α and C/EBPα knockdown

**Figure 3:**
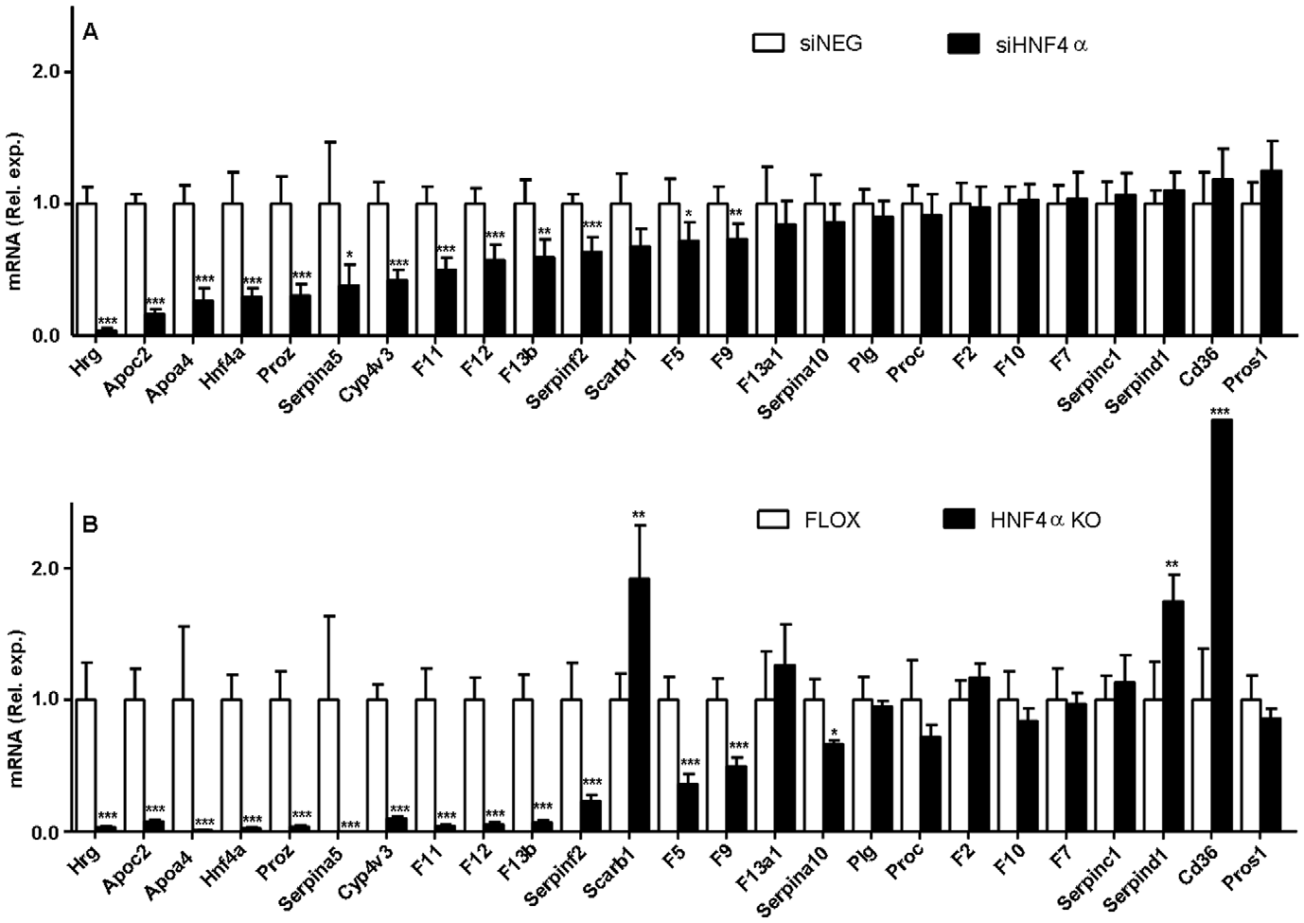
Comparison of gene transcript levels in mouse livers following HNF4α knockdown and liver-specific targeted deletion of *Hnf4a*. Livers from siHNF4α injected animals (panel A, black bars) and siNEG injected animals as controls (panel A, open bars) were subjected to control and coagulation gene transcript levels by QPCR. In panel B, coagulation transcript analysis for liver materials from HNF4α-null mice (KO) or control mice (FLOX) is included for comparison [25]. Data are presented for mice two days post siRNA injection or app. 6 weeks gene ablation (i.e. 6 weeks old HNF4α-null mice). β-actin was used as internal control for quantification and normalization. The ΔCt values of the individual samples were related to the mean ΔCt of the siNEG or FLOX control group. Data are expressed as mean with error bars representing the difference between 2 POWER of upper and lower range of the mean ΔΔCt (see figure 1 legends). Data were statistically analysed using Mann Whitney Rank Sum test. *P*-values<0.05 were regarded as statistically significant. *P<0.05, **P<0.01, ***P<0.001. On the x-axis the coagulation genes are ranked according to the magnitude of effects observed in the siRNA injected animals.

**Figure 4:**
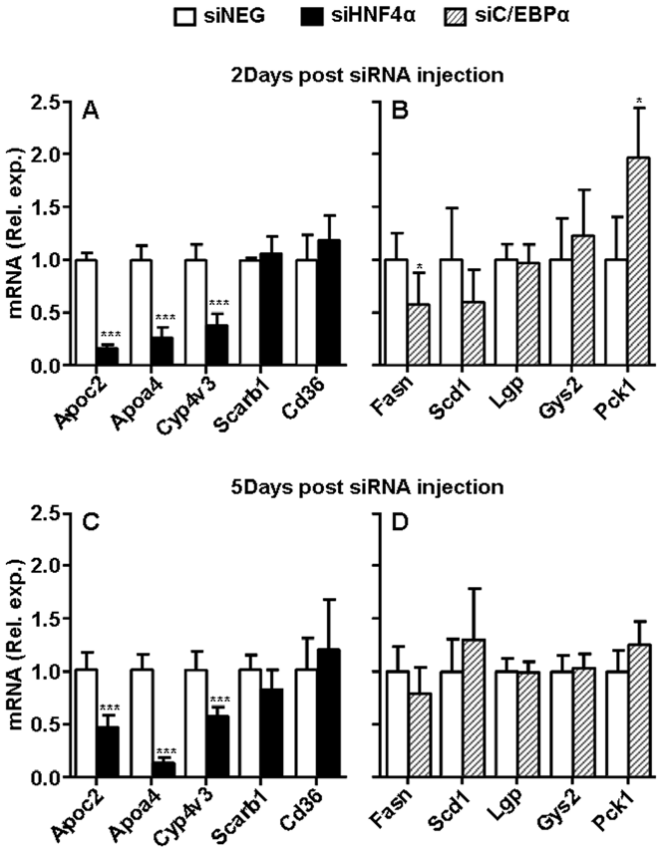
Hepatic transcription of control genes following HNF4α and C/EBPα knockdown. At two (upper panels) and five days (lower panels) post siRNA injection, mouse livers were subjected to transcript levels by QPCR for a panel of control genes. Data are presented for siHNF4α (panel A and C, black bars) and siCEBPα (panel B and D, hatched bars) with siNEG injected animals as controls (panel A, B, C and D, open bars). β-actin was used as internal control for quantification and normalization. The ΔCt values of the individual samples were related to the mean ΔCt of the siNEG group. Data are expressed as mean with error bars representing the difference between 2 POWER of upper and lower range of the mean ΔΔCt (see figure 1 legends). Data and were statistically analysed using Mann Whitney Rank Sum test. *P*-values<0.05 were regarded as statistically significant. *P<0.05, ***P<0.001.

**Figure 5:**
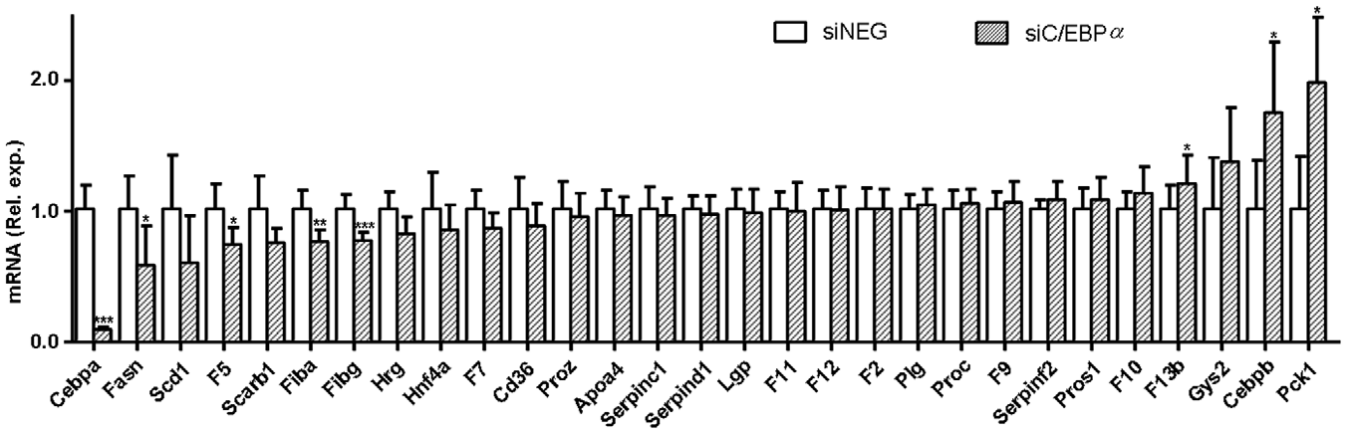
Overview of gene transcript levels in mouse livers following C/EBPα knockdown. Livers from siCEBPα injected animals (hatched bars) and siNEG injected animals as controls (open bars) were subjected to control and coagulation gene transcript levels by QPCR. Data are presented for mice two days post siRNA injection. β-actin was used as internal control for quantification and normalization. The ΔCt values of the individual samples were related to the mean ΔCt of the siNEG group. Data are expressed as mean with error bars representing the difference between 2 POWER of upper and lower range of the mean ΔΔCt (see figure 1 legends). Data were statistically analysed using Mann Whitney Rank Sum test. *P*-values<0.05 were regarded as statistically significant. *P<0.05, **P<0.01, ***P<0.001. On the x-axis the coagulation genes are ranked according to the magnitude of effects observed in the siRNA injected animals.

In livers of 6-wk-old liver-specific HNF4α deficient animals [25] apolipoprotein C2 (*Apoc2*), apolipoprotein A4 (*Apoa4*), and cytochrome P450 family member 4v3 (*Cyp4v3*) are among the genes that are highly expressed in the liver and are strongly down-regulated by prolonged HNF4α ablation, whereas in these mice scavenger receptor B1 (*Scarb1*) and CD36 (*Cd36*) are among the genes that are strongly upregulated [25], [34] (Figure 3B). The upregulation is suspected to be secondary and may result of prolonged hepatic HNF4α ablation. In livers of mice two days post siHNF4α injection, like in livers of liver-specific HNF4α deficient mice, strongly reduced *Apoc2*, *Apoa4* and *Cyp4v3* transcript levels were observed (Figure 4A). Also at five days, *Apoc2*, *Apoa4*, and *Cyp4v3* remained significantly reduced in siHNF4α compared to the siNEG injected animals, despite the limited level of HNF4α knockdown at this time point (Figure 4C and 2A). *Scarb1* and *Cd36* transcript levels were not affected by siHNF4α injection at two or five days (Figure 4A and 4C). Mice in which hepatic C/EBPα has been targeted by recombinant adenovirus encoding siRNA against C/EBPα mRNA demonstrated a role in (fasted) liver glucose and fat metabolism by affecting amongst others transcription of phosphoenolpyruvate carboxykinase (*Pck1*), glycogen synthase (*Gys2*), fatty acid synthase (*Fasn*), stearoyl-CoA-desaturase 1 (*Scd1*) and liver glycogen phophatase (*Lgp*) [32]. As demonstrated in Figure 4B, two days post siC/EBPα injection, the livers of (non-fasted) mice displayed significant changes in transcript levels of *Pck1* and *Fasn*, *Scd1* displayed a 40% non-significant decrease, whereas *Gys2* and *Lgp* were unaffected as compared to siNEG injected animals. As in C/EBPα knockouts [35], siC/EBPα injection resulted in a significant increase in transcript levels of *Cebpb* (+73%, Figure 5) which is considered a compensatory response to C/EBPα knockdown [35]. *Cebpb*, *Pck1*, *Fasn* and *Scd1* transcript levels in siC/EBPα-injected mice were comparable to levels in siNEG animals at five days after injection, despite the persistent strong level of knockdown (Figure 2B and 4D).

### Changes in liver coagulation gene transcription following HNF4α and C/EBPα knockdown

**Figure 6:**
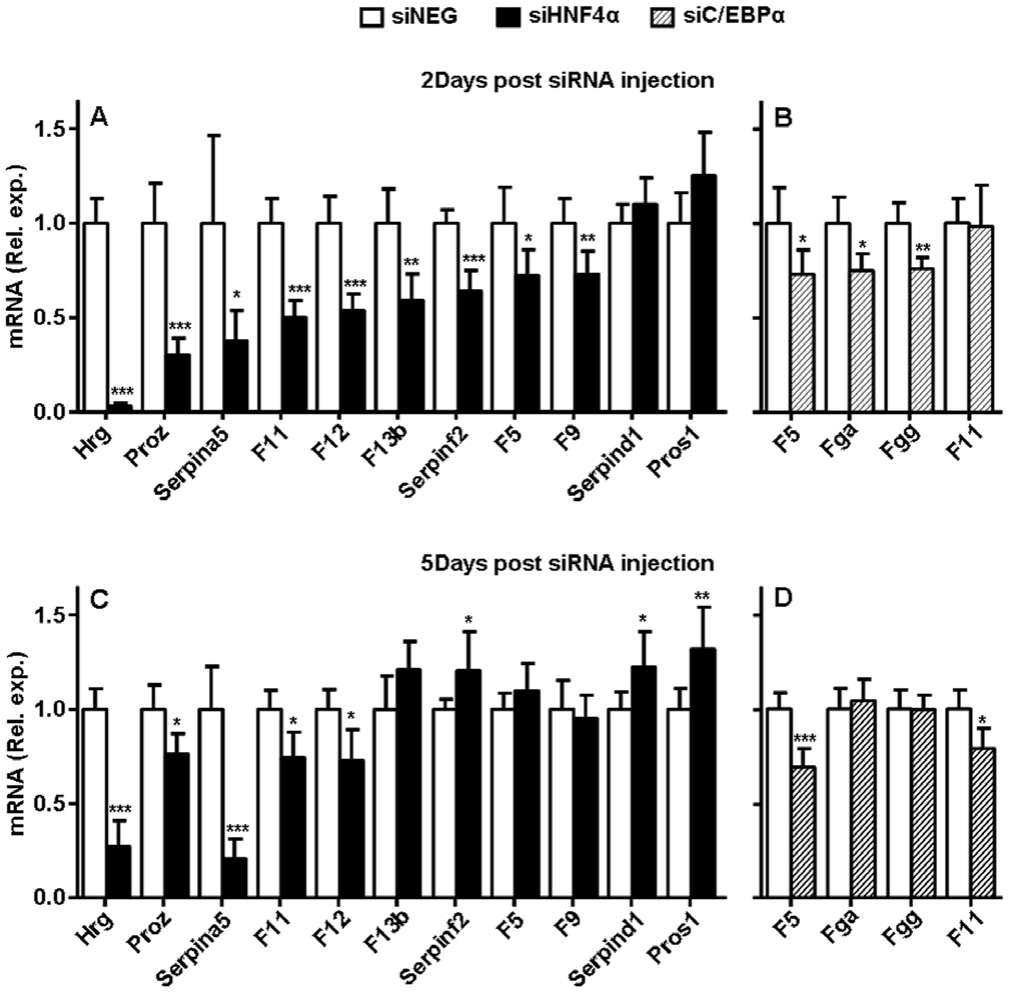
Hepatic coagulation gene transcription following HNF4α and C/EBPα knockdown. At two (upper panels) and five days (lower panels) post siRNA injection, mouse livers were subjected to coagulation gene transcript levels by QPCR. Data are presented for siHNF4α (panel A and C, black bars) and siCEBPα (panel B and D, hatched bars) with siNEG injected animals as controls (panel A, B, C and D, open bars). β-actin was used as internal control for quantification and normalization. The ΔCt values of the individual samples were related to the mean ΔCt of the siNEG group. Data are expressed as mean with error bars representing the difference between 2 POWER of upper and lower range of the mean ΔΔCt (see figure 1 legends). Data were statistically analysed using Mann Whitney Rank Sum test. *P*-values<0.05 were regarded as statistically significant. *P<0.05, **P<0.01, ***P<0.001. Only those coagulation genes are presented for which transcript levels were significantly affected by siHNF4α or siC/EBPα (in two or five days study group). On the x-axis the coagulation genes are ranked according to the magnitude of effects observed in the two day groups.

Livers of siHNF4α injected animals with strong reduction in HNF4α transcript and protein levels (two days) displayed markedly and significantly reduced transcript levels of *Hrg* (−97%), *Proz* (−70%), *Serpina5* (−62%), *F11* (−50%), *F12* (−46%), *F13b* (−41%), *Serpinf2* (−36%), *F5* (−38%), *F9* (−27%) (Figure 6A). For the other coagulation genes that were analysed (Figure 3A) we did not observe significant changes as compared to siNEG injected animals. At five days, transcript levels of *Hrg*, *Proz*, *Serpina5*, *F11* and *F12* (−73%, −24%, −79%, −26%, and −27%, respectively) remained reduced, despite the limited level of HNF4α knockdown at this time point (Figure 2A, 6C). Interestingly, at five days, significant elevations in transcript levels of *Serpinf2*, *Serpind1* and *Pros1* (+20%, + 22%, +32%, respectively) were observed (Figure 6C). Overall, the HNF4α-mediated downregulation of coagulation gene transcript levels in livers of siHNF4α injected animals seemed to largely reproduce those observed in 6 week old HNF4α-null mice with prolonged HNF4α ablation in the liver from birth on (compare Figure 3A, 3B), albeit at a lower extent and, importantly, with two-day siHNF4α injected animals not showing any upregulating effects.

Compared to HNF4α, C/EBPα knockdown had a more modest impact on coagulation gene transcription. Strong reduction in C/EBPα transcript and protein levels resulted in small though significantly reduced transcript levels of fibrinogen α and γ (*Fga* and *Fgg* −25% and −24%, respectively) and *F5* (−27%) at two days post siC/EBPα injection. Only, the reduction in *F5* transcript levels persisted upon prolonged C/EBPα knockdown (−31%). At this time point also *F11* transcript levels became significantly reduced (−21%). For the many other coagulation genes that were analyzed, neither at two (Figure 5) nor at five days (data not shown) post siC/EBPα injection, we observed changes as compared to siNEG injected animals that reached statistical significance.

At two days, plasma from siHNF4α and siC/EBPα injected animals had an APTT and PT comparable to siNEG injected animals (Table 2). Although analyzed for only a limited number of individual coagulation factors, at this time point, HNF4α knockdown coincided with a significant reduction in plasma F11 activity levels, but not F12 activity levels. C/EBPα knockdown coincided with significant reduction of plasma fibrinogen antigen but not F5 activity levels. At five days, siHNF4α injected animals displayed a minimal but significant shortening of the PT, while that of siC/EBPα injected animals was minimally but significantly prolonged. Plasma fibrinogen antigen levels remained reduced upon prolonged C/EBPα knockdown.

## Discussion

In the present study we used an *in vivo* synthetic small interfering RNA approach to determine in mice the direct role of the liver transcription factors hepatocyte nuclear factor 4α (HNF4α) and CCAAT/enhancer-binding protein α (C/EBPα) in coagulation gene transcription under conditions minimizing methodology or target-related secondary effects. Shortly (two days) post intravenous siHNF4α injection, we observed (strong) reductions in transcript levels of the *Hrg, Proz, Serpina5, F11, F12, F13b, Serpinf2, F5, and F9* genes indicating that these coagulation genes are under direct regulatory control of HNF4α. The relatively modest but fast reduction in transcript levels of *Fga*, *Fgg* and *F5* observed two days post-siC/EBPα injection indicating that these genes likely targets for direct regulatory control by C/EBPα. However, siC/EBPα injection also rapidly induced increased C/EBPβ transcription, indicating a rapid onset of C/EBPα-related secondary (compensatory) effects and therefore we cannot exclude an underestimation of C/EBPα's direct role in coagulation gene transcription. Overall, we conclude that in the mouse, HNF4α has a direct and essential regulatory role for multiple hepatic coagulation genes. For C/EBPα, such a role is more restricted, but may be underestimated as result of an unexpectedly fast compensatory upregulation of *Cebpb* transcription.

Analyzing the impact of liver HNF4α deficiency on a genome-wide scale using microarrays identified an estimated 20% of the hepatically expressed genes affected by HNF4α deficiency to be upregulated [32], [34]. *Scarb1* and *Cd36* were among the genes that were strongly upregulated in liver-specific HNF4α deficient mice while unaffected upon acute siRNA-mediated HNF4α knockdown in mice. It was speculated that *Scarb1* increase may be a secondary consequence of altered lipid homeostasis (e.g., due to changes in intracellular lipid levels in liver of HNF4α-null mice) [25]. A similar mechanism may account for *Cd36* albeit that this gene is predominantly in the liver Kupffer cells. The absence of any statistically significant upregulating effects regarding transcription in livers of mice two days post siHNF4α injection, including *Scarb1* and *Cd36*, suggests that at least a large portion of the upregulating effects observed in the liver-specific knockouts are secondary to prolonged HNF4α disruption. This supports the use of our siRNA approach for *in vivo* studies on the regulating role of liver transcription factors. In this light, it is worthwhile to denote that prolonged knockdown i.e. 5 days post siHNF4α, despite a limited efficacy of hepatic HNF4α knockdown (Figure 2A and 2D), resulted in likely secondary upregulating effects for a number of the coagulation genes (*Serpinf2*, *Serpind1*, *Pros1*, Figure 6C).

In the present study, C/EBPα was selected as the second liver transcription factor in this first synthetic siRNA study on transcriptional control of liver coagulation genes given the observations from a recent CHIPseq study on the genome-wide occupancy of C/EBPα in livers of multiple species [16]. In this study, fibrinogen A (FGA) and prothrombin (F2) were among the few genes (from a total of 32 in mouse genome) that were located near ultraconserved C/EBPα binding regions. Indeed, in our mouse study we identified *Fga* as one of the few genes likely to be under direct transcriptional control of C/EBPα. However, *F2* was clearly not affected following siC/EBPα, suggesting that the C/EBPα binding site located 64 bp from the transcription start site in the mouse *F2* promoter is not critical for physiological control of *F2* transcription. In addition, we were surprised not to find an effect of siC/EBPα on mouse hepatic *F9* transcription. In humans, carriers of hemophilia B Leiden have a causal mutation in a C/EBPα binding site in F9 promoter [17], and in line, mice with prolonged C/EBPα ablation the liver display reduced hepatic expression of F9 [18]. Possibly, C/EBPα interaction with the *F9* promoter, and also that of *F2*, is of high affinity, requiring only limited levels of C/EBPα binding to drive transcription. Despite the strong level C/EBPα reduction of transcript (−92% and −87% at 2 and 5 days respectively) and protein levels (Figure 2B and 2D) by our synthetic siRNA approach this may not be sufficient to unmask a role for C/EBPα for these type of targets. Alternatively, the observed unexpectedly rapid upregulation of C/EBPβ transcript following siC/EBPα injection, may functionally replace C/EBPα in liver [36]. Indeed, C/EBPβ has been shown to compensate for loss of C/EBPα in the regulation of *Pck1* gene expression [37]. Thus, provided the C/EBPβ transcript rapidly translates to protein (which we did not determine) a compensation for loss of C/EBPα by C/EBPβ cannot be excluded and may explain the absence of effects of siC/EBPα on F2 and F9 and possibly other genes.

The *in vivo* siRNA delivery procedure used had low toxicity without effects on circulating liver enzymes tested (Table 2) as expected [28]). However, as compared to the control siRNA, both siHNF4α and siC/EBPα had a mild transient effect on serum alkaline phosphatase levels, the circulating marker for biliary obstruction. This suggests the presence of target-related (mild) hepatotoxic effects. Alternatively, changing in ALP levels may reflect a specific transcriptional regulatory role of HNF4a and C/EBPa for genes involved in regulation of bile acid biosynthesis, as has been reported for HNF4α [25]. In the present study, whether siHNF4α or C/EBPα had an immediate effect on bile acid biosynthesis genes like *Cyp7a1*, *Cyp27a1* and *Cyp8b1* was not investigated.

Negative control siRNAs - siRNAs with sequences that do not target any gene products - are essential to control for the effects of siRNA delivery, and to determine whether an siRNA is considered to have a positive, negative, or neutral effect in a particular assay. In our animal studies, we included a commercially available negative control siRNA that was designed to have no significant sequence similarity to mouse, rat, or human transcript sequences (for description see methods and [29]). This negative control siRNA incorporates the same chemical modifications and is purified to the same rigorous specifications as the target-specific siRNAs (siHNF4α and siCEBPα). In addition, this negative control siRNA virtually lacked effects on gene transcription as determined for multiple cell lines following exposure of relatively high doses of negative control siRNA and analyzed by whole genome expression arrays. Despite the careful design of the negative control, we cannot exclude that in our experiments the control siRNA itself had influence on our genes of interest, and thereby leading to misinterpretation of the findings. To fully exclude such misinterpretation, in vivo experiments should be expanded with multiple carefully designed negative and multiple target specific siRNA. The HNF4α-mediated (downregulatory) changes in control and coagulation genes in livers of siHNF4α injected animals largely reproduced that of 45-day-old HNF4α-null mice and wild-type mouse primary hepatocytes rapidly after siRNA-mediated HNF4α knockdown, indicating that the single negative control siRNA approach used in the present study allowed reliable estimation of the (direct) effects of siHNF4α and also that of siC/EBPα.

*In vitro* siRNA screening and validation in (primary) mouse hepatocytes showed that the siRNAs targeting HNF4α had higher efficacy than those targeting C/EBPα (Figure 1). Remarkably, *in vivo* we observed the opposite i.e. the siC/EBPα were more effective than siHNF4α (Figure 2). Both HNF4α and C/EBPα displayed normal and stable expression in primary mouse liver cells i.e Ct comparable to fresh livers. This suggests that the discrepancy between the *in vitro* and *in vivo* findings could not be attributed to a rapid decline in HNF4α *in vitro* and thereby the efficacy of the siHNF4α is over estimated. Whether the difference in efficacy is due to differences in *in vivo* siRNA delivery, processing and or stability of the siRNA is unknown. However, it emphasizes that the *in vitro* experiments (as performed) are useful for identifying siRNAs with *in vivo* potential, but are not fully predictive for identifying most effective siRNA for *in vivo* use. Although, these aspects of *in vivo* siRNA approach should be improved, this study demonstrates that synthetic siRNA provides a simple and fast means for determining direct transcription factor involvement *in vivo* under conditions minimizing secondary effects. Here, *in vivo* siRNA-mediated knockdown enabled us to establish the direct contribution of HNF4α and C/EBPα to the regulation of coagulation gene transcription.
